# Outcome assessment of different reward stimuli in Internet gaming disorder by event-related potentials

**DOI:** 10.1371/journal.pone.0307717

**Published:** 2024-07-24

**Authors:** Mengyue Zhang, Chenyue Zhao, Ziyu Mao, Meng Zhang, Xiaoli Xing

**Affiliations:** Department of Psychology, Henan University, Kaifeng, Henan, China; Yale University, UNITED STATES OF AMERICA

## Abstract

An imbalance in sensitivity to different types of reward stimuli may be an important cause of addiction that is mainly manifested in high sensitivity to addictive substance rewards and blunting of natural rewards. However, contradictions remain in the research results on the sensitivity of individuals with Internet gaming disorder (IGD) to different reward stimuli. Based on participants’ neural responses to win and loss feedback (in door task), the event-related potential (ERP) technique was used to investigate the effects of different types of reward stimuli on the assessment of reward-processing outcomes in individuals with IGD. The results showed that in the gain condition, the FB-P3 amplitude induced by game stimuli in the IGD group was significantly higher than that in the control group, and the FN amplitude induced by money stimuli was significantly lower than that in the control group. However, the FB-P3 and FN amplitudes induced by food were not significantly different from those in the control group. In the loss condition, there were no between-group differences in the FB-P3 and FN amplitudes evoked by the three reward stimuli. This indicates that the IGD group showed increased hedonic responses to game stimuli and decreased hedonic responses to money but no differences in hedonic responses to food during the reward assessment phase. Therefore, heightened sensitivity to game rewards and diminished sensitivity to monetary rewards during outcome assessment may play a crucial role in the development of IGD.

## Introduction

As a rewarding stimulus, moderate Internet game use brings relaxation and pleasure, whereas excessive Internet game use can bring about a series of negative effects, such as lower subjective well-being and more negative emotions, including unhappiness and loneliness [1,2]. A meta-analysis study showed that the current global prevalence of Internet gaming disorder (IGD) in adolescents and young adults is as high as 9.9%, making it one of the most common mental disorders [3]. It refers to pathological game-using behavior in which an individual seeks physical and psychological pleasure in Internet games and is unable to control their behavior. In 2022, Internet Gaming Disorder was included in the Fifth Edition of Diagnostic and Statistical Manual of Mental Disorders Text Revision (DSM-V-TR) as a condition for further study. The prevalence of IGD and its great detrimental effects have made it a serious mental health problem worldwide [4].

An imbalance in sensitivity to different types of reward stimuli may be a major cause of addiction. For individuals with addiction, reward triggers can be categorized as addictive and non-addictive material rewards, with addictive material rewards including addictive substances and addiction-related stimuli [5]. Non-addictive material rewards, also known as natural rewards, include primary and secondary rewards. Primary rewards are considered necessary for species survival [6] and include reward cue conditions such as water and food, which are relevant to people’s organismal states. Secondary rewards are not directly related to survival and are values obtained through acquired reward-habitual associations, such as money, power, and other reward cue conditions [7]. Studies on many types of addiction have found that individuals with addiction have increased sensitivity to addictive substances and blunted natural rewards. For example, individuals with nicotine addiction (substance addiction) have a specific response to tobacco [8] and a blunted response to food rewards [9,10]. Individuals with gambling addiction (behavioral addiction) are also highly sensitive to gambling cues and have a smaller network of responses evoked by food rewards [11].

Reward stimulus processing is divided into two phases: reward anticipation and outcome assessment. Although the two phases have different neural control mechanisms, they interact with each other. Reward anticipation refers to the emotional and motivational states associated with craving production, in which dopamine signaling plays an important role [12,13] and is associated with reward anticipation in the bilateral anterior insula, ventral striatum, and brainstem regions [14]. Outcome assessment refers to the hedonic experience of reward acquisition [15], a phase in which endogenous opioids and cannabinoids play an important role [16], and the nucleus ambiguus, ventral anterior cingulate gyrus, medial orbitofrontal cortex, and amygdala are associated with the outcome assessment of reward [17]. These two processes interact, with the dopaminergic signaling involved in both the reward anticipation and outcome assessment stages. In the reward anticipation phase, dopamine activity is correlated with the predictive value of reward cues, and a reward-related higher probability of predictive cues induces a greater degree of dopamine neuron activation [18]. During the reward outcome assessment phase, dopamine encodes the difference between the actual reward outcome received and the expected outcome [18,19]. The substantia nigra and ventral tegmental area of the midbrain sends error signals to relevant cortical structures to adjust reward expectations as well as subsequent decisions and behaviours [20,21]. Thus, players with internet gaming addiction gain greater motivational value from reward-related cues by anticipating error signals each time they play a game.

Reward processing is continuous in the temporal dimension, and thus this process can be effectively captured using event-related potential (ERP) techniques with high temporal resolution. In the reward anticipation phase, the stimulus-preceding negativity (SPN), a negative component located in the center of the frontal lobe that usually occurs 200 ms before feedback, can be used as a reliable indicator of reward expectancy, reflecting the level of an individual’s expectation of an upcoming reward or punishment [22]. In the outcome assessment phase, Feedback Negativity (FN) and Feedback P3 (FB-P3) are two important ERP components. FN is a negative deflection that occurs in the frontal center approximately 250–350 ms after the appearance of a feedback stimulus and is an electrophysiological indicator of reward sensitivity in response to reward, particularly the discrepancy between expectation and outcome [23]. In addition, FB-P3 is a positive ERP component that appears in the central-parietal lobe 300–600 ms after the appearance of the feedback stimulus. Unlike Cue-P3, FB-P3 reflects the allocation of attentional resources to information related to the efficacy of the outcome [24] as well as affective processes by conveying the motivational salience of reward feedback.

Much of the current IGD-related reward processing research has focused on the anticipatory phase, which is characterized by increased craving for substance-addictive stimulus game play, decreased craving for the primary reward stimulus, food, and no significant change in craving for the secondary reward stimulus, money. Individuals with IGD have been found to have an attentional bias toward game stimuli as well as increased craving [25,26] but potentially impaired food reward craving. Individuals with IGD showed a significantly higher craving response when responding to game cues than to food cues, and game-related cues caused a significantly greater increase than food-related cues in the precuneus lobe-caudate nucleus relationship, eliciting higher functional connectivity [26,27]. In terms of secondary rewards, monetary rewards did not appear to be impaired during the reward anticipation phase in individuals with IGD. In a simple gambling task, the SPN amplitude that induced IGD during money choice was not significantly different from that of the control group [15]. Also, there was no difference in neural network activity between the addicted and control groups during the anticipation of reward outcomes by the money gain/loss cue [28].

Relatively little attention has been paid to the mechanisms of outcome assessments in individuals with IGD in response to different rewarding stimuli. Individuals with IGD have been found to have enhanced responses to game stimuli in neuroimaging studies. This is evidenced by increased response strength to gaming-related cues in reward regions of the brain, as well as heightened activation in regions associated with emotional arousal, including the orbitofrontal cortex, nucleus accumbens, and anterior cingulate gyrus [26,29]. In contrast, individuals with IGD showed attenuated responses to monetary secondary reward stimuli. In a simple gambling task, individuals with IGD showed a decrease in P3 and Feedback Negativity (FN) amplitudes, both of which are event-related potentials (ERPs), following an increase in reward, indicating a decrease in sensitivity to monetary attentional resources. FN reflects an individual’s reward sensitivity, and P3 reflects the allocation of participants’ attentional resources [30]. FMRI results have also shown that players with internet gaming addiction have reduced striatal activation during unexpected monetary rewards [31] and that other brain regions associated with assessment (right caudate nucleus, left orbitofrontal cortex, and dorsolateral prefrontal cortex) were less activated than in controls [32]. Reduced sensitivity to monetary reward outcomes is an important characteristic of individuals with IGD, which suggests that they have difficulty processing secondary rewards. This may lead to an increased reliance on virtual rewards in games to make up for the lack of real-life access to rewards, thus exacerbating their dependence on games [33,34]. However, monetary stimuli activate more areas of the preorbital frontal cortex associated with hedonic responses in individuals with pathological gambling issues than do primary reward stimuli, such as food and sex [35]. This may be because money is directly associated with the acquisition of gambling chips, which they exchange on a daily basis, whereas the IGD population are primarily time contributors. Additionally, it has been found that individuals who smoke have diminished activation in the insula, nucleus accumbens, inferior frontal cortex, and lateral lid in response to pictures of desired food, suggesting a reduced neural response to natural rewards [36]. However, it is unclear how individuals with IGD are sensitized to food during the outcome assessment phase, and it is possible that individuals with IGD may also exhibit reduced responses to food rewards.

The different responses of players with internet gaming addiction during the two processes of reward suggest that there may be different cognitive neural bases for addiction. The present study focuses on the brain mechanisms of the three types of reward stimuli, game, food, and monetary stimuli, during the outcome assessment phase. There are two reasons for this. First, these three types of reward stimuli represent different types of important reward stimuli and play a central role in the prediction of human social behavior. In IGD, gaming stimuli are addictive stimuli, whereas food stimuli are the most common primary reward stimuli in humans. Money, which is associated with a variety of human needs such as food, security, power, and reputation, is the most common and important secondary reward for humans, and its responses predict an individual’s social behavior [7]. Second, there have been no direct comparative studies of the assessment phase of the outcomes of the three types of reward stimuli. According to the reward deficiency syndrome (RDS) theory, individuals with addiction have dopamine reward circuits that are under-activated for natural rewards; therefore, they are driven to seek strong reward experiences to compensate for the lack of reward feelings [35]. Thus, individuals with IGD may become addicted to Internet gaming to obtain the missing “pleasure” by compensating for the under-activation of the midbrain dopamine path in response to reward signals. Therefore, individuals with IGD may underreact to natural rewards and overreact to game rewards during the outcome assessment phase.

## Materials and methods

### Participants

Current college students were recruited through questionnaires distributed in offline classes as well as a professional platform named “Wenjuanxing” for data collection questionnaire surveys and screened against the inclusion and exclusion criteria (all participants were familiar with Glory of Kings). The participants had normal or corrected vision, signed an informed consent form, and were paid a certain amount of money after completing the experiment. They were also asked to fast for two hours before the experiment.

The inclusion criteria for the IGD group were as follows: (1) scores of ≥ 5 on the DSM-V, (2) scores of ≥ 50 on the adapted version of the Internet Addiction Test (IAT) [38], (3) continuous gaming for at least 2 years and more than 14 hours of gaming per week in the last year, and (4) gaming accounted for more than 50% of the daily Internet activities. Playing games accounted for more than 50% of their daily Internet activities. Inclusion criteria for health control (HC) were as follows: (1) score < 5 on the DSM-V, (2) score < 50 on the adapted version of the IAT; (3) had not played games in the last year or not more than 2 hours per week, and (4) games accounted for less than 20% of daily Internet activities. The exclusion criteria were a history of neurological disorders, mental illnesses such as depression, former or current drug dependence, alcohol or tobacco addiction, and other types of behavioral addictions such as gambling.

A total of 45 participants met the requirements; five participants with excessive head movements and artifacts were excluded to improve the accuracy and reliability of the experimental data, and the final sample consisted of 20 individuals in the IGD group (4 females, 16 males) and 20 individuals in the control group (13 females, 7 males). The mean age was 19.35 years, with a standard deviation of 0.48 years. None of the participants had a history of neurological disease or brain injury, and they were free of depression in the previous week (score less than 16 on the depression scale used by the Flow Control Center).

The research protocol of this study was approved in writing by the Ethics Committee of the Key Laboratory of Psychology and Behaviour in Henan Province. All procedures were performed in accordance with the Declaration of Helsinki. Participant recruitment for this study begins on 15 October 2022 and ends on 25 October 2022.

### Experimental design

The study used a 3 (reward type: food reward, monetary reward, game reward) × 2 (feedback type: win, lose) × 2 (group: control group, addiction group) mixed analysis of variance (ANOVA), in which the dependent variable was the mean amplitude of the ERP components (FN, FB-P3). The FN reflects sensitivity to outcome-expectation discrepancies, and the FB-P3 reflects the allocation of attentional resources to information related to outcome validity. These two components can well reveal the differences in brain activity between the two groups when processing different rewards and feedbacks. Participants were asked to complete three tasks, food, monetary, and game-door tasks, in which the group was the between-subjects factor, and feedback type and reward type were the within-subjects factors. The order of the tasks between the participants was counterbalanced according to the Latin square.

### Experimental materials

Game usage questionnaire: Some basic investigations of the subject’s gaming behavior, such as the number of hours of gaming per week in the past 12 months, the percentage of gaming behavior among total Internet use, gaming history, the three games specifically played the most in the past 12 months, the average number of hours of weekly use for each game, and tobacco and alcohol use.

The Internet Gaming Addiction Questionnaire (Diagnostic and Statistical Manual of Mental Disorders-5, DSM-5): This questionnaire is based on the nine diagnostic criteria of the DSM-V [4]. Participants were asked to answer questions based on their internet gaming activities in the past 12 months. The response options for each item include “yes = 1” and “no = 0”. Each item corresponds to a symptom of Internet gaming addiction, and a score of 5 or more meets the DSM-V screening criteria for internet gaming addiction.

The adapted version of the Internet Addiction Test (IAT): This study used an adapted version of the Internet Addiction Test developed by Young [37,38]. It consists of 20 items measured on a 5-point Likert scale, ranging from “1 = never” to “5 = always”. It focuses on assessing the amount of time individuals have spent playing games in the past month and the negative impact of gaming on their lives [31,38]. A score of 50 or higher indicates a tendency to become addicted to Internet games.

The Game Craving Scale: The scale is based on the Questionnaire on Gaming Urges Brief (QGU-B), which was adapted from the Questionnaire on Smoking Urges Brief (QSU-B) developed by Cox, Tiffany and Christen [39,40]. The scale comprises 10 items where participants rate whether they currently experience the situation described in each item. The response options for each item are “yes = 1” and “no = 0”, with higher scores indicating a stronger craving for the game.

The experimental stimuli were ice cream symbols, monetary symbols, and game symbols (monetary symbols were represented by "¥" and game symbols by the game logo of Glory of Kings). To ensure the validity of the materials, 25 Glory of Kings gamers were recruited to rate pleasantness (from 0 = "very calm" to 5 = "very pleasant") and familiarity (from 1 = "I am not familiar at all" to 5 = "I am very familiar") in relation to the symbols of the food representations, monetary symbols, and game symbols. Finally, the validity of the selected pictures was analyzed, and there were no significant differences among the three pictures in the assessment of pleasantness (food: M±SD = 4.240±0.156; monetary: M±SD = 4.280±0.158; games: M±SD = 3.720±0.187, *p* > 0.050), nor in the assessment of familiarity (food: M±SD = 4.320±0.160; monetary: M±SD = 4.720±0.108; games: M±SD = 4.360±0.128, *p* > 0.050). These 25 students did not participate in the formal experiment.

### Experimental tasks and procedures

**Fig 1:**
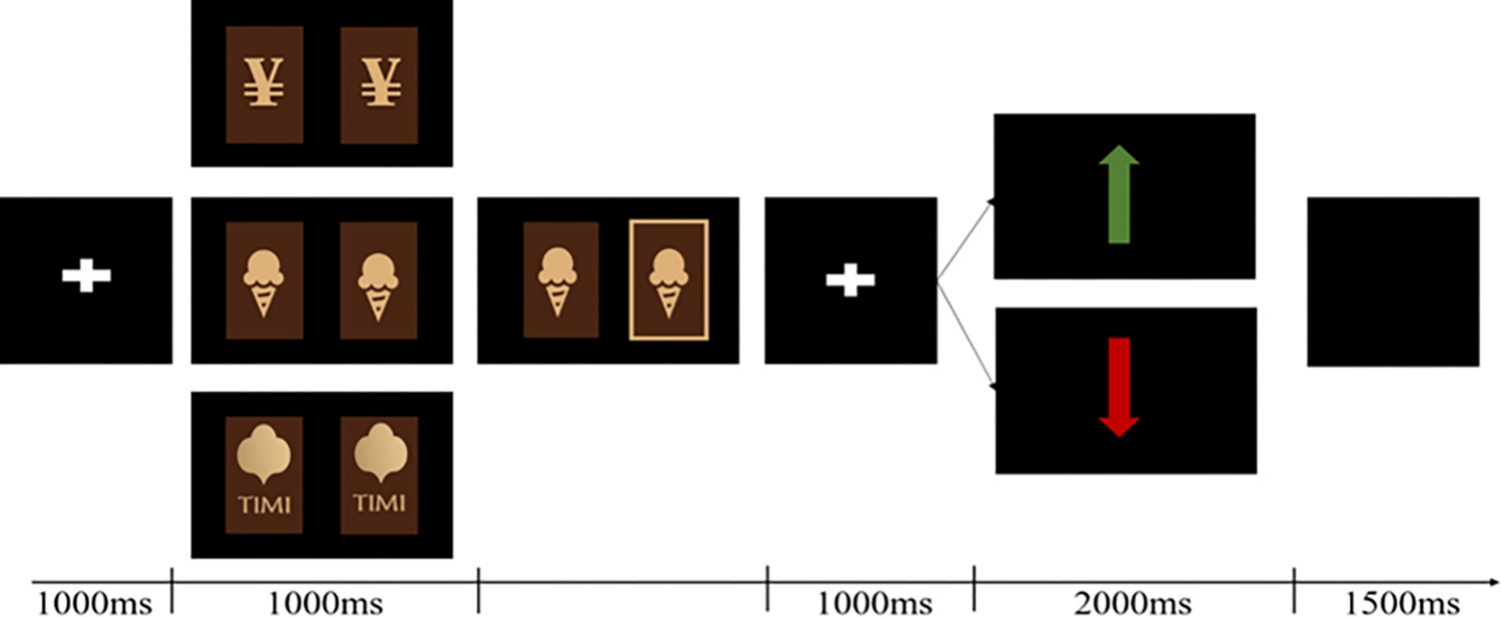
Door task trial flow.

In the door task, "food,” "monetary,” and "game" were presented as stimuli in block form and in Latin square order. Feedback was represented as "meaningless" images (up/down arrows). The experiment was programmed using E-prime 3.0. The door task was a forced-choice guessing task in which participants were informed before beginning the task that they would have the opportunity to win a reward. In each trial, participants were presented with two doors and asked to press either the "f" or "j" key to select the door that they thought hid the reward. After making their choice, they were prompted with the appropriate feedback. The green "up arrow" indicated a reward, meaning that the participant won 50 snack points/$5/50 coupons, and the red "down arrow" indicated a loss, meaning that the participant lost 25 snack points/$2.50/25 coupons, with win and loss feedback each presented 50% of the time in a randomized order. Each trial consisted of the following sequence: After the gaze point "+" was presented for 1000ms, an image of two doors was presented, and the participant pressed "f" or "j" to select one to open; the selected door was presented continuously for 1000ms, followed by the presentation of the gaze point "+" for 1000ms, the feedback result for 2000ms, and a blank screen for 1500ms, as shown in Fig 1. The formal experiment had three blocks of 50 trials each, with 25 each for gains and losses.

### EEG data acquisition

Based on the experimental total average waveform graphs and previous related studies, the FN (220-300ms) and FB-P3 (280-400ms) EEG components were selected as observational indices after presenting the feedback results. The FN was included in the statistical analysis with an average waveform amplitude of 220–300ms after the appearance of feedback, and the corresponding electrode positions were Cz, FC2, and FCz. FB-P3 was included in the statistical analysis, with an average wave amplitude of 280–420ms after the emergence of feedback; the corresponding electrode positions were Cz, Pz, CP1, and CP2 [41].

### Statistical analysis

In this study, SPSS 22.0 was used for data analysis. The Greenhouse-Geisser method was employed to correct the results of ANOVA, and the Bonferroni method was used to correct the results of multiple comparisons.

## Results

### Statistics on basic information of participants

An independent samples t-test was conducted on the self-report questionnaire data and the scores of the Game Craving Scale. A chi-square test was performed on the sex ratio between the two groups. The preliminary results of the analyses are detailed in Table 1. The data analysis showed that there was no significant difference between the two groups (control group, addiction group) in terms of age, and there was a significant difference in scores on the DSM-5 and The Internet Addiction Test as well as in number of hours of gameplay per week and game craving, *p* < 0.001.

**Table 1:** Statistics on basic information of participants.

Variant	Addiction group	Control group	*p*
Gender ratio (females:males)	4:16	13:7	= 0.004
Age	19.35±0.98	19.77±1.87	0.373
DSM-5	6.10±1.04	0.83±1.02	<0.001
The adapted version of the IAT	57.38±9.14	28.00±6.79	<0.001
Hours of play per week	22.04±11.48	1.43±1.52	<0.001
Game craving	3.52±3.01	0.82±0.83	<0.001

### ERP results

#### FN component

The 3 (reward type: food reward, monetary reward, game reward) × 2 (feedback: win, lose) × 2 (group: control group, addiction group) repeated-measure ANOVA showed that the difference between groups was not significant (*F*_1, 38_ = 0.33, *p* = 0.568, *η*^*2*^_*p*_ = 0.01). Reward type was also not significant (*F*_2, 76_ = 0.22, *p* = 0.796, *η*^*2*^_*p*_ = 0.01), while the main effect of feedback type was significant (*F*_1, 38_ = 10.25, *p* = 0.003, *η*^*2*^_*p*_ = 0.21).

The group and reward type interaction was significant (*F*_2, 76_ = 9.98, *p* < 0.001, *η*^*2*^_*p*_ = 0.20), while neither the group and feedback type interaction (*F*_1, 38_ = 0.66, *p* = 0.420, *η*^*2*^_*p*_ = 0.01) nor the reward type and feedback type interaction were significant (*F*_2, 76_ = 2.78, *p* = 0.068, *η*^*2*^_*p*_ = 0.06).

The three-way interaction was significant (*F*_2,76_ = 4.53, *p* = 0.014, *η*^*2*^_*p*_ = 0.10). Simple effects analyses at the level of group showed that wave amplitudes for monetary feedback was higher for the control group in the win condition (5.46 ± 1.06 μV) than for food (3.37 ± 0.99 μV, *p* = 0.001) and the game stimuli (3.73 ± 1.01 μV, *p* = 0.030). Additionally, the control group showed higher wave amplitudes in the monetary and game winning conditions (5.46 ± 1.06 μV; 3.73 ± 1.01 μV) than in the losing condition (3.49 ± 0.91 μV, *p* < 0.001; 2.46 ± 0.86 μV, *p* = 0.042). In the winning condition, the addicted group’s wave amplitudes were higher for the game (4.27 ± 1.01 μV) than for monetary stimuli (2.18 ± 1.06 μV, *p* = 0.007), and in the losing condition, the amplitude of the waves evoked by the addicted group to the game (3.26 ± 0.82 μV) was slightly higher than the amplitude to the money cue (1.94 ± 0.91 μV, *p* = 0.069). Simple effects analyses with reward type as a level found that the control group evoked significantly higher wave amplitudes for monetary stimuli (5.46 ± 1.06 μV) than the addicted group (2.18 ± 1.06 μV, *p* = 0.036) in the win condition, while the two groups were not significantly different in the food and game conditions. In the loss condition, there was no significant difference in the amplitude of waves evoked by the two groups in the three reward stimulus conditions (*p* > 0.05).

#### FB-P3 component

The 3 (reward type: food reward, monetary reward, game reward) × 2 (feedback: win, lose) × 2 (group: control group, addiction group) repeated-measure ANOVA showed that the difference between groups was not significant (*F*_1, 38_ = 0.50, *p* = 0.482, *η*^*2*^_*p*_ = 0.01). The main effect of reward type was not significant (*F*_2, 76_ = 0.16, *p* = 0.848, *η*^*2*^_*p*_ = 0.00), while the main effect of feedback type was significant (*F*_1, 38_ = 5.10, *p* = 0.030, *η*^*2*^_*p*_ = 0.11).

The interaction between group and reward type was significant (*F*_2, 76_ = 6.01, *p* = 0.004, *η*^*2*^_*p*_ = 0.13), while the interaction between the group and feedback type was non-significant (*F*_1, 38_ = 0.04, *p* = 0.830, *η*^*2*^_*p*_ = 0.00). Additionally, the difference between reward type and feedback type was non-significant (*F*_2,76_ = 0.02, *p* = 0.975, *η*^*2*^_*p*_ = 0.00).

The three-way interaction tended to be significant (*F*_2,76_ = 3.05, *p* = 0.053, *η*^*2*^_*p*_ = 0.07). Simple effects analyses at the level of group found that wave amplitudes were higher for monetary (4.65 ± 0.96 μV) than for gaming stimuli in the gain condition (2.98 ± 0.86 μV, *p* = 0.007) and they were higher in the monetary gain condition (4.65 ± 0.96 μV) than in the monetary loss condition (3.78 ± 0.92 μV, *p* = 0.013). The addicted group had higher wave amplitudes for game (5.42 ± 0.86 μV) than for monetary stimuli in the gain condition (3.90 ± 0.96 μV, *p* = 0.016) and for food gain than for food loss (3.74 ± 0.81 μV, *p* = 0.051). A simple effects analysis with reward type as the level found that the addiction group evoked higher waveforms under game gains (5.42 ± 0.86 μV) than the control group (2.98 ± 0.86 μV, *p* = 0.053). In the loss condition, there was no statistically significant difference in wave amplitudes between the two groups across the three reward stimulus conditions (p > 0.05).

**Fig 2:**
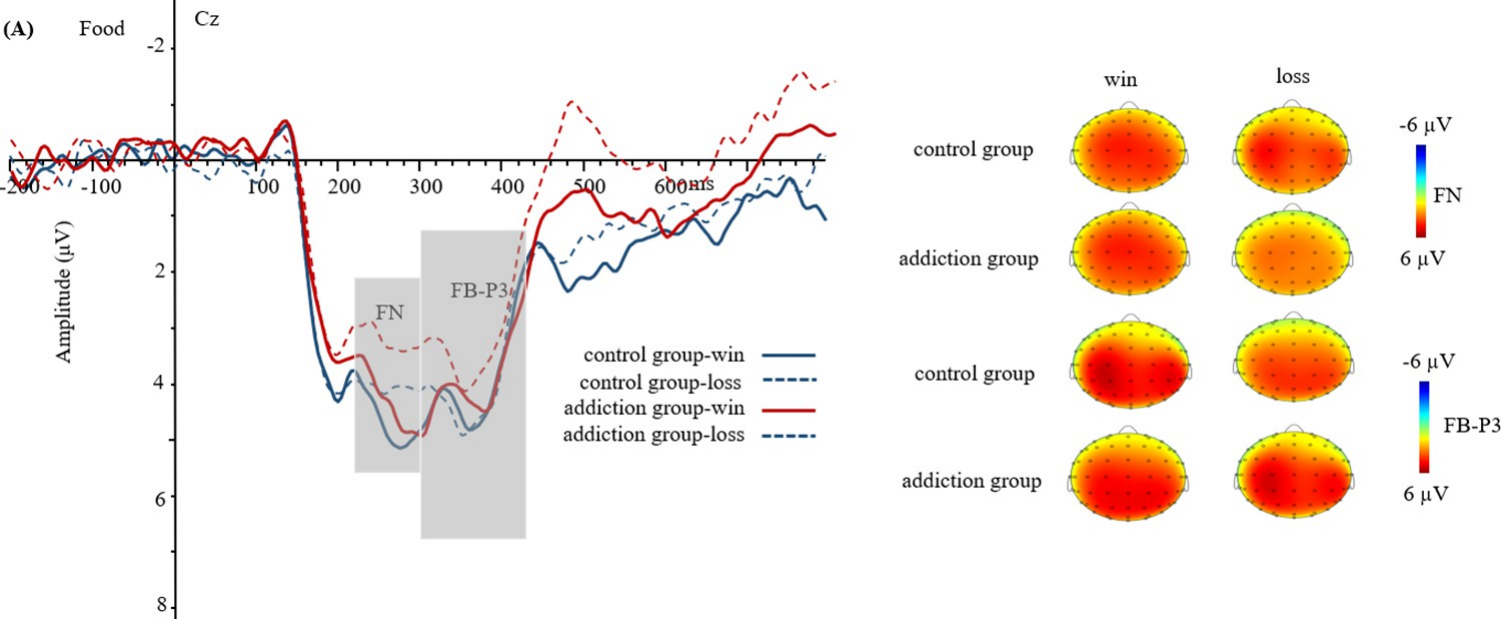
Grand-average event-related potential (ERP) waveforms and topographic maps in food reward (at point Cz).

**Fig 3:**
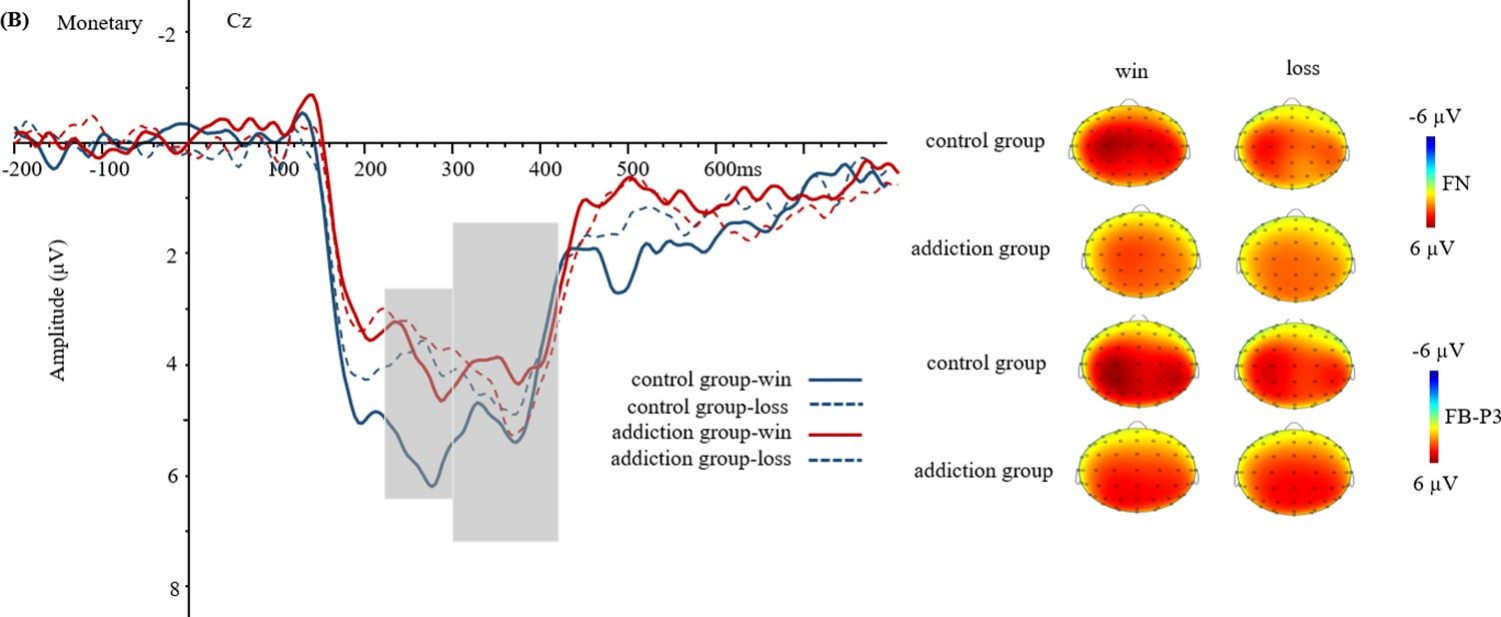
Grand-average event-related potential (ERP) waveforms and topographic maps in monetary reward (at point Cz).

**Fig 4:**
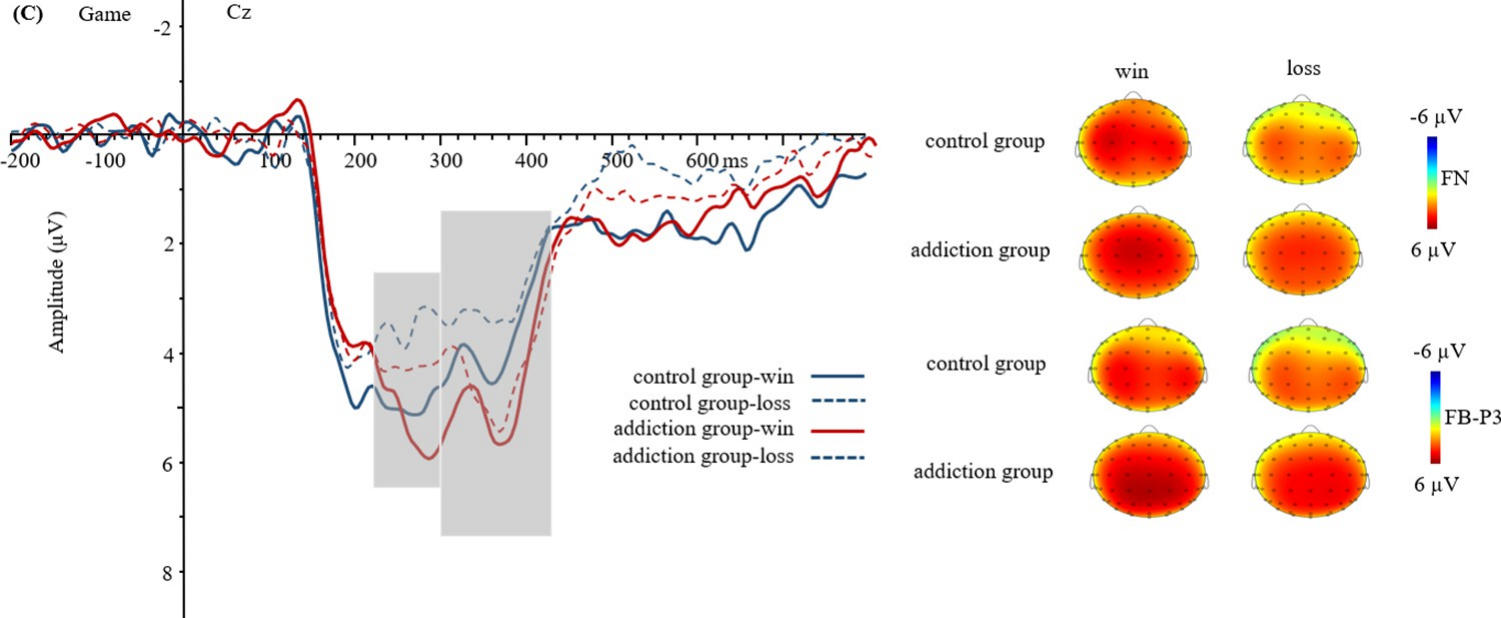
Grand-average event-related potential (ERP) waveforms and topographic maps in game reward (at point Cz).

The waveforms and topography of FN and FB-P3 in food reward are shown in Fig 2. The waveforms and topography of FN and FB-P3 in monetary reward are shown in Fig 3. The waveforms and topographic maps of FN and FB-P3 in game reward are shown in Fig 4.

The EEG results of the two groups in response to the three types of reward stimuli are summarized in Table 2.

**Table 2:** EEG results of responses to three types of rewarding stimuli between the two groups.

Group/reward type		Electroencephalographic (EEG) component	
		FN	FB-P3
Group	Control group	Monetary > food, game	Monetary > game
	Addiction group	Game > monetary	Game > monetary
Reward type	Food reward	No between-group differences	No between-group differences
	Monetary reward	Control group > addiction group	No between-group differences
	Game reward	No between-group differences	Addiction group > control group

## Discussion

The present study explored the imbalance in sensitivity to different types of rewards in individuals with IGD through an experimental approach combined with the event-related potential technique based on the outcome assessment phase using a door task. The results preliminarily confirmed that individuals with IGD have an enhanced hedonic response to gaming stimuli and a reduced hedonic response to monetary rewards but no difference in their hedonic response to food in the outcome assessment phase.

The IGD group exhibited an enhanced response to game gains compared to the control group. That is, in the door task, the IGD group evoked significantly higher FB-P3 wave amplitudes in response to game gains than the control group. Reward mechanisms may evoke positive emotions in players, whereas punishment mechanisms may evoke negative emotions. One study observed changes in BOLD responses in the dorsolateral prefrontal cortex and hippocampus during video game training, and these changes were specifically associated with reward-related stimuli in the game environment [42]. Another study investigating the effects of different reward scenarios on game-related cognition and behavior showed that winning scenarios were more stimulating and reduced players’ boredom and frustration [43]. In addition, the "NEAR MISS" effect, which refers to the feeling of being close to winning, may also be a reason why individuals with IGD are more sensitive to game reward feedback. Research suggests that “coming up short” fosters gambling behavior [44] and triggers activity in the brain’s reward centers (i.e., dopaminergic pathways). In line with this, a study on video games also found that participants experienced a stronger urge to continue playing and more frustration when they experienced an “almost” event [45]. Thus, the NEAR MISS effect may enhance players’ expectation of and sensitivity to game rewards by activating dopaminergic pathways, causing players to continually pursue winning experiences. These studies demonstrate that feedback in games elicits strong emotional responses from individuals, which further influences players’ choice to continue playing. In the case of Glory of Kings, for example, its one attractive feature is its feedback mechanism, in which the game provides players with timely feedback from the beginning to the end of the game, including screen presentations and voice announcements, so that the players are reinforced. This may be the reason why IGD individuals are highly sensitive to game feedback.

The IGD group exhibited a decreased response to monetary gain relative to the control group. In the door task, the IGD group evoked significantly lower FN amplitudes in response to monetary gains than the control group, which is consistent with previous studies. An electrophysiological study reported that adolescents with Internet gaming addiction had reduced FN amplitudes after receiving monetary rewards compared to a control group [30]. Similarly, in a study on nicotine addiction, when researchers compared tobacco and monetary feedback stimuli separately, they found that individuals with nicotine addiction evoked greater FN amplitudes in the tobacco feedback condition [8]. Thus, individuals with addiction experience greater FN when processing addictive substances than when processing monetary rewards. In conclusion, reduced sensitivity to monetary feedback during the outcome assessment phase appears to be a common feature among different addictions [46]. Addictive behaviors may be caused by a reduced ability to monitor reward feedback and difficulty in adjusting excessive gaming behaviors based on feedback outcomes. In the reinforcement learning theory, FN represents the reward prediction error signal. Abnormalities in the FN in individuals with IGD in response to monetary feedback may also be a blunted response to reward prediction signals, and blunted sensitivity to monetary rewards may diminish the effects of monetary reward reinforcement, thereby potentially impairing a person’s reinforcement learning [32] and making it difficult for addictive individuals to derive pleasure from everyday life.

In the present study, the increased sense of gaming feedback and decreased sensitivity to monetary feedback in individuals with IGD can be explained by the pleasure deficit syndrome model of addiction [35]. This theory proposes that the dopamine reward circuitry of individuals with addiction may have deficits in the activation of naturally rewarding behaviors, resulting in an individual being in a state of pleasure deficit, whereas addictive substances or behaviors would give the addict a higher sense of pleasure. As a result, individuals may engage in repetitive gaming behaviors in search of pleasure, and frequent gaming may lead to specific alterations in the reward system of these individuals, which may raise their reward threshold, making it more difficult for everyday rewards to activate the reward system of individuals with addiction.

Sensitivity to food rewards did not change in the IGD group relative to the control group. That is, there were no significant differences in the FN and FB-P3 wave amplitudes induced by the addiction and control groups in the food reward condition in the door task. The results of the present study did not verify our hypothesis or support previous findings [10,47,48]. This may have been because the food stimuli selected for the experiment were not sufficiently attractive. Firstly, the food stimuli chosen in this experiment were symbols rather than images. Symbols (e.g., words or simple signs) differ in triggering cognitive and emotional experiences compared to images, and visual stimuli (e.g., images and photographs) better mimic real-world experiences, and can trigger emotional responses and attention more directly and strongly [49]. For example, in cognitive tasks, pictures elicited stronger attentional interference effects than words [50]. This suggests that symbols may not be emotionally evocative enough to effectively stimulate participants’ attention and cravings, which could potentially explain the lack of significant change in their sensitivity to food rewards during the experiment. Second, sex differences may have contributed to the insensitivity to food rewards in the addicted group. In general, individuals with IGD are predominantly male, which was also the case in this study [51]. Studies have shown that men are more interested in meal-related high-calorie foods such as pizza, pasta, steak, and beef, whereas women have a preference for snack-related comfort foods such as candy and chocolate [52]. Therefore, the ice cream stimuli chosen may not have elicited sufficient interest and response in male individuals with IGD. In addition, the neural response to food stimuli is stronger in women than in men in brain regions such as the parahippocampal gyrus, thalamus, and precuneus, which have been shown to have relevance in promoting eating [53]. This gender difference may be one of the reasons why sensitivity to food rewards remains constant in individuals with IGD.

## Limitations and future research directions

The present study has several limitations. First, more groups could be included for comparison to draw more reasonable conclusions. The current study group consisted mostly of individuals with IGD and individuals with normal gaming use, while recreational players fall in between these two and may exhibit similar or different characteristics. Second, future research should enrich different types of reward stimuli. Studies have shown that IGD and depression are highly co-morbid, and individuals with these issues are socially under-motivated, implying that their social reward processing may be abnormal. Therefore, it is necessary to further differentiate among the different types of reward stimuli in subsequent studies.

## Conclusion

In the reward outcome assessment phase, the IGD group showed an increased hedonic response to game stimuli and a decreased hedonic response to money, but no difference in hedonic response to food. Heightened sensitivity to game rewards and diminished sensitivity to monetary rewards during outcome assessment may play a crucial role in the development of IGD.

## Supporting information

S1 Appendix Game usage questionnaire. (DOCX)

S2 Appendix The internet gaming addiction questionnaire (diagnostic and statistical manual of mental Disorders-5, DSM-5). (DOCX)

S3 Appendix Adapted version of the internet addiction test. (DOCX)

S4 Appendix The game craving scale. (DOCX)

S5 Appendix FN average amplitude. (XLSX)

S6 Appendix FB-P3 average amplitude. (XLSX)
