# Supplementary material for: Outcome assessment of different reward stimuli in Internet gaming disorder by event-related potentials
Source: PLoS One. 2024 Jul 24;19(7):e0307717. doi: 10.1371/journal.pone.0307717 (PMC11268701; doi:10.1371/journal.pone.0307717)
Supplement: S1 Appendix — (DOCX) [file pone.0307717.s001.docx]

**Appendix S1**

**Game usage questionnaire**

The following is the questionnaire about the basic personal information and the frequency of daily use of the game, we will ensure the privacy of the results of each student questionnaire, please choose truthfully according to the actual situation in the past 12 months!

1. Gender (male, female)

2. age

3. height , weight

4. preferred hand (left-handed, right-handed)

5. vision (normal vision, colour vision, colour blindness)

6. years from the first time you played the game continuously (including playing every now and then) to the present ( ) years (if less than a year, you can directly fill in months, for example, three months)

7. In the past year, your favourite game is ( ), and you play games for ( ) hours a day, or ( ) hours a week on average.

8. How much time you spend on the Internet playing games? (e.g. 50%)

9. How many cigarettes do you smoke per day on average?

10. in public places where smoking is prohibited, e.g. classrooms, libraries, cinemas, etc., do you find it difficult because you are not allowed to smoke: (Yes, No)

11. How often do you drink alcohol: (never, once a month, 2-4 times a month, 2-3 times a week, more than 4 times a week)
