# Supplementary material for: Outcome assessment of different reward stimuli in Internet gaming disorder by event-related potentials
Source: PLoS One. 2024 Jul 24;19(7):e0307717. doi: 10.1371/journal.pone.0307717 (PMC11268701; doi:10.1371/journal.pone.0307717)
Supplement: S2 Appendix — (DOCX) [file pone.0307717.s002.docx]

**Appendix S2**

**The Internet Gaming Addiction Questionnaire (Diagnostic and Statistical Manual of Mental Disorders-5, DSM-5)**

Please answer the following questions based on your internet gaming activity in the last 12 months. The internet gaming we refer to here includes all forms of gaming behaviour such as online gaming, single player gaming, and so on.

| 1.Do you spend a lot of time thinking about games even when you are not playing, or planning when you can play next? | Yes | No |
| --- | --- | --- |
| 2.Do you feel restless, irritable, moody, angry, anxious or sad when attempting to cut down or stop gaming, or when you are unable to play? | Yes | No |
| 3.Do you feel the need to play for increasing amounts of time, play more exciting games, or use more powerful equipment to get the same amount of excitement you used to get? | Yes | No |
| 4.Do you feel that you should play less, but are unable to cut back on the amount of time you spend playing games? | Yes | No |
| 5.Do you lose interests in or reduce participation in other recreational activities (hobbies, meetings with friends) due to gaming? | Yes | No |
| 6.Do you continue to play games even though you are aware of negative consequences, such as not getting enough sleep, being late to school/work, spending too much money, having arguments with others, or neglecting important duties? | Yes | No |
| 7.Do you lie to family, friends or others about how much you game, or try to keep your family or friends from knowing how much you game? | Yes | No |
| 8.Do you game to escape from or forget about personal problems, or to relieve uncomfortable feelings such as guilt, anxiety, helplessness or depression? | Yes | No |
| 9.Do you risk or lose significant relationships, or job, educational or career opportunities because of gaming? | Yes | No |
