# Supplementary material for: Outcome assessment of different reward stimuli in Internet gaming disorder by event-related potentials
Source: PLoS One. 2024 Jul 24;19(7):e0307717. doi: 10.1371/journal.pone.0307717 (PMC11268701; doi:10.1371/journal.pone.0307717)
Supplement: S3 Appendix — (DOCX) [file pone.0307717.s003.docx]

**Appendix S3**

**Adapted version of the Internet Addiction Test**

The following questionnaire consists of 20 statements. Please read the statement carefully and select an answer (from 1 to 5) that best describes your situation during the past month. 1 means ‘Never’, 2 means ‘Occasionally’, 3 means ‘Frequently’, 4 means ‘Often’, and 5 means ‘Always’. Please read all the statements carefully before making your choice.

|  | Never | Occasionally | Frequently | Often | Always |
| --- | --- | --- | --- | --- | --- |
| 1. How often do you find that you play online games longer than you intended? | 1 | 2 | 3 | 4 | 5 |
| 2. How often do you neglect household chores to spend more time on online games? | 1 | 2 | 3 | 4 | 5 |
| 3. How often do you prefer the excitement of the Internet to intimacy with your gaming partners? | 1 | 2 | 3 | 4 | 5 |
| 4. How often do you form new relationships with other game players? | 1 | 2 | 3 | 4 | 5 |
| 5. How often do others in your life complain to you about the amount of time you spend on online games? | 1 | 2 | 3 | 4 | 5 |
| 6. How often do your grades or school work suffer because of the amount of time you spend on online games? | 1 | 2 | 3 | 4 | 5 |
| 7. How often do you play online games before something else that you need to do? | 1 | 2 | 3 | 4 | 5 |
| 8. How often does your job performance or productivity suffer because of the online games? | 1 | 2 | 3 | 4 | 5 |
| 9. How often do you become defensive or secretive when anyone asks the time you spend on games? | 1 | 2 | 3 | 4 | 5 |
| 10. How often do you block out disturbing thoughts about your life with soothing thoughts of the online games? | 1 | 2 | 3 | 4 | 5 |
| 11. How often do you find yourself anticipating when you will play games again? | 1 | 2 | 3 | 4 | 5 |
| 12. How often do you fear that life without the online games would be boring, empty, and joyless? | 1 | 2 | 3 | 4 | 5 |
| 13. How often do you snap, yell, or act annoyed if someone bothers you while you are playing online games? | 1 | 2 | 3 | 4 | 5 |
| 14. How often do you lose sleep due to playing online games? | 1 | 2 | 3 | 4 | 5 |
| 15. How often do you feel preoccupied with the online games when off-line, or fantasize about being playing games? | 1 | 2 | 3 | 4 | 5 |
| 16. How often do you find yourself saying "just a few more minutes" when you play games? | 1 | 2 | 3 | 4 | 5 |
| 17. How often do you try to cut down the amount of time you spend on online games and fail? | 1 | 2 | 3 | 4 | 5 |
| 18. How often do you try to hide how long you've played online games? | 1 | 2 | 3 | 4 | 5 |
| 19. How often do you choose to spend more time on online games over going out with others? | 1 | 2 | 3 | 4 | 5 |
| 20. How often do you feel depressed, moody or nervous when you are off-line, which goes away once you are back to games? | 1 | 2 | 3 | 4 | 5 |
