# Supplementary material for: Outcome assessment of different reward stimuli in Internet gaming disorder by event-related potentials
Source: PLoS One. 2024 Jul 24;19(7):e0307717. doi: 10.1371/journal.pone.0307717 (PMC11268701; doi:10.1371/journal.pone.0307717)
Supplement: S4 Appendix — (DOCX) [file pone.0307717.s004.docx]

**Appendix S4**

**The Game Craving Scale**

Please read each of the following questions carefully and make your choice based on the option that best suits your personal circumstances by ticking the appropriate option

| 1. I have a desire to play games right now | Yes | No |
| --- | --- | --- |
| 2. Nothing would be better than playing games right now | Yes | No |
| 3. If it were possible, I might be playing a game right now | Yes | No |
| 4. I could control myself better now if I could play games | Yes | No |
| 5. All I want to do now is play games | Yes | No |
| 6. I have a strong desire to play games | Yes | No |
| 7. It would be nice to play games now. | Yes | No |
| 8. I would do anything to play games now | Yes | No |
| 9. Games would make me less depressed | Yes | No |
| 10. I am going to play games as soon as possible | Yes | No |
